# Supplementary material for: Eupalinolide J Inhibits Cancer Metastasis by Promoting STAT3 Ubiquitin-Dependent Degradation
Source: Molecules. 2023 Mar 31;28(7):3143. doi: 10.3390/molecules28073143 (PMC10096386; doi:10.3390/molecules28073143)
Supplement: Supplementary file 1 [file molecules-28-03143-s001.zip › molecules-2236088-supplementary.pdf]

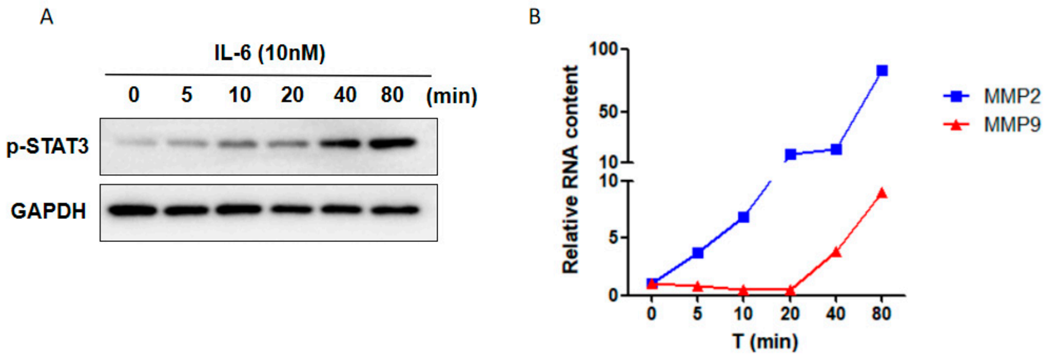

Figure S1. STAT3 activation induced the expression of MMP-2 and MMP-9 mRNA in MDA-MB-231 cells. A. IL-6 induces STAT3 activation in MDA-MB-231 cells; B. Changes of MMP-2 and MMP-9 mRNA over time in MDA-MB-231 cells after STAT3 activation.
